# Are We Eating Our Way to Prostate Cancer—A Hypothesis Based on the Evolution, Bioaccumulation, and Interspecific Transfer of miR-150

**DOI:** 10.3390/ncrna2020002

**Published:** 2016-04-21

**Authors:** Venkatesh Vaidyanathan, Vetrivhel Krishnamoorthy, Nishi Karunasinghe, Anower Jabed, Radha Pallati, Chi Hsiu-Juei Kao, Alice Wang, Gareth Marlow, Lynnette R. Ferguson

**Affiliations:** 1 Discipline of Nutrition and Dietetics, FM & HS, University of Auckland, Auckland 1023, New Zealand; vkri468@aucklanduni.ac.nz (V.K.); rpal628@aucklanduni.ac.nz (R.P.); b.kao@auckland.ac.nz (C.H.-J.K.); alice.wang@auckland.ac.nz (A.W.); l.ferguson@auckland.ac.nz (L.R.F.); 2 Auckland Cancer Society Research Centre, Auckland 1023, New Zealand; n.karunasinghe@auckland.ac.nz; 3 Department of Molecular Medicine and Pathology, FM & HS, University of Auckland, Auckland 1023, New Zealand; a.jabed@auckland.ac.nz; 4 Experimental Cancer Medicine Centre, Cardiff University, Cardiff, CF14 4XN, UK; MarlowG@cardiff.ac.uk

**Keywords:** miR-150, evolution, nutrigenomics, prostate cancer, bioaccumulation

## Abstract

MicroRNAs (miRNAs) are well established epigenetic modifiers. There is a lot of work being done to identify the evolutionary transfer of miRNAs both at intra- and interspecific levels. In this hypothesis-driven review, we have suggested a possible reason as to why miR-150 can be a promising diagnostic biomarker for prostate cancer using theories of evolution, bio-accumulation, and interspecific transfer of miRNAs.

## 1. Introduction

Epigenetic modifications are well known to play vital roles in oncogenesis [1,2]. A key regulator of epigenetic change is micro-ribonucleic acid (miRNA) [3]. miRNAs are not only post-transcriptional gene expression modifiers [2], but miRNAs also have been identified to play roles in DNA methylation processes [2] and affect the expression of enzymes involved in the metabolic pathways of DNA methylation [4]. Conversely, methylation also plays an important role in the expression of miRNAs [5,6]; miRNAs also affect chromatin structures by regulating histone modifications [7]. These small, non-coding single stranded nucleic acids were first discovered in the nematode, *Caenorhabditis elegans* in 1993 [8], but only in recent times have miRNAs been identified to be influential in various different aspects and fields of research, especially as diagnostic and progressive biomarkers for human diseases [9], including cancer [10,11], especially prostate cancer (PCa) [12]. The 22 nucleotide hairpin loop structure of miRNA is vital in its role as a post-translational modifier, allowing it to bind to messenger ribonucleic acid (mRNA), thereby preventing protein translation [13].

PCa is a complex disease which originates in the glandular cells of the prostate. It has one of the highest incidence rates amongst all diagnosed cancers in males worldwide [14,15]. Predicting the aggressiveness of this cancer is crucial for patient outcomes, including chances of survival and quality of life. Based on various factors such as prostate-specific antigen (PSA) level, and/or pre-operative Gleason score, and/or clinical stage, the aggressiveness of PCa is graded [16]. It is of utmost importance to identify individuals with aggressive PCa, as this will not only improve the lifestyle of the patients with non-aggressive PCa, but also help identify an early treatment regime for the patients with the aggressive form of disease. 

**Figure 1:**
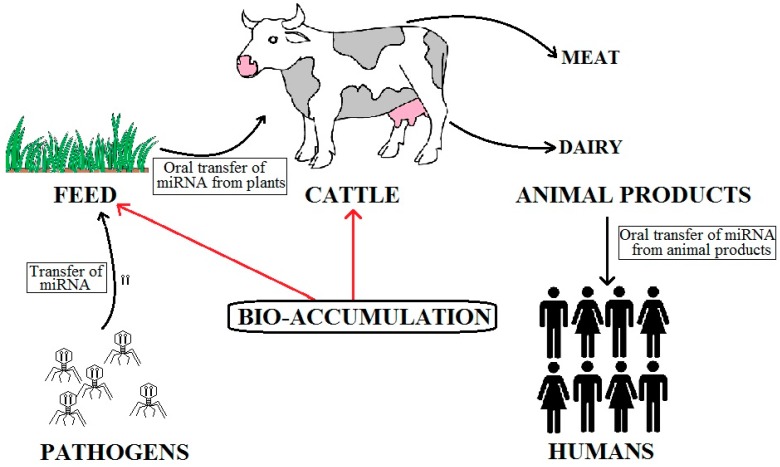
Possible entry route of miR-150 from pathogens to humans. Since bio-accumulation of miR-150 occurs at two steps, and that different forms of animal products are consumed by humans, it is vital to identify the levels of miR-150 that can be transferred and required for the normal being of humans. Also, amongst human, the transfer of miR-150 may take place from males to females and back to offspring through the process of reproduction, such that miR-150 can be used as a diagnostic and therapeutic biomarker for PCa.

miRNAs have been shown to be sensitive to external factors such as physical activity and diet [17], thereby raising the possibility of their influence on varied metabolic responses [18]. Evolutionary history also contributes crucial factors that influence the pathology of human diseases [19]. Thus we looked into the aspect of evolution, interspecific transfer, and the influence of external factors such as food and the role of certain miRNAs to identify if there is a link between what we eat and PCa aggressiveness. Our working hypothesis is summarized in Figure 1.

## 2. miR-150 and Cancer

miRNA has oncogenetic activity when altered by mechanisms like deletions, mutations and amplifications [20] causing dysregulation of genomic expression and interactions. miR-150 is a circulating miRNA which indicates that it is active and moves through serum plasma, allowing it to be widely distributed and hence readily available from bodily fluids such as blood [21]. The accumulation and over expression of miR-150 was initially correlated with cancer, and multiple articles have indicated its potential role as a biomarker for breast cancer [22], cervical cancer [23], colorectal cancer [24], lung cancer [25,26], chronic lymphocytic leukemia [27], and metastatic PCa [28,29]. The expression levels of miR-150 were, however, significantly down-regulated in the pancreatic ductal adenocarcinoma tissues compared to the benign pancreatic tissues adjacent to it [30].

## 3. miR-150: A Potential Biomarker for Prostate Cancer

PCa is currently diagnosed on a population scale subsequent to abnormal screening results of the PSA level and/or digital rectal examination; however, these measures do little to accurately and objectively analyze and determine the extent of potential danger a patient is in. Metastatic PCa is a condition where cells are liberated from normal regulatory mechanisms allowing the cancer to spread unchecked resulting in potentially lethal patient outcomes. While benign neoplasms are common with minor symptoms, it is still difficult to distinguish between the two without an accurate, objective preliminary measure. miR-150 is known to be elevated in metastatic PCa and can provide an objective, accurate view of a patient’s well-being, allowing for better outcomes [28].

Waltering *et al.*, 2011, reported that miR-150* along with three other miRNAs, namely, miR-10a, miR-141, and miR-1225-5p, among seventeen miRNAs, expressed to have >1.5- fold changes at the expression levels in PCa cells and in a minimum of two intact-castration xenograft pair [31]. It has been shown that miR-150 was overexpressed in PCa cells [28]. One of the other key findings by the same group was that the patients with high expression levels of miR-150 had a tendency towards developing tumor recurrence [28]. Increased miR-150 expression may influence the expression of PCa by the suppression of p27- a cyclin-dependent kinase inhibitor [29]. In mammals, interestingly, the expression profile of miR-150 most likely underlies the regulatory processes of hormonal events or changes. The menstruation cycle among female mammals is an example of such an event [32]. In women, Rekker *et al.* in 2013 identified miR-150 in the plasma profile during menstruation [33]. This pattern is consistent with the miRNA expression profile of ovulating cattle as well, whereby miR-150 becomes progressively more accumulated with progression of the estrous cycle, especially on day 7 [34,35]. Therefore, it can be inferred that steroid hormones, which are key players in mammalian menstruation, are associated with oncosis [36].

Cattle, particularly cows, are often treated with hormones [37] to induce reproductive mechanisms and potentially increase yields of milk [38] as well as fertility [39]. These additional female sex hormones are often of much concern for the well-being of human consumers of these products [40]. Moreover, phytoestrogens are established hormone-like compounds that are well known to be partially secreted into breast milk creating a bioaccumulative effect [41].

As described previously, miR-150 can potentially be bioaccumulated in oncogenesis. Steroid hormones like estrogens and androgens could therefore be upregulated as a result of miR-150 bioaccumulation. The involvement of estrogen and its receptors is already established as a risk factor in cancer development [40], particularly in the context of epithelial to mesenchymal transitions (EMTs) [42]. The EMT is implicated in the process of metastasis [43], which, with respect to miR-150 explains its accuracy as a biomarker that is indicative of patient mortality [28]. 

In this manuscript, we have suggested a possible route of entry of miR-150 into humans, and raised the possibility that this could be associated with harmful effects from the consumption of animal products such as meat, milk and dairy products.

## 4. Past and Present Genome Scenario

Evolution requires a trait to be advantageous and heritable [44]. miRNAs have many diverse subtypes and hence biological functions. In order to have established themselves as vital epigenetic modifiers with such variation, miRNAs have evolved over time [45,46]. Early miRNAs are hypothesized to have arisen as an immune response to viral infection (acting on protein synthesis) [47] and even now, they are considered as part of the human immune system (for example, miR-155) [48]. Since their origin, miRNAs, based on their function, have diversified considerably [46]. For example, miR-1 has modulatory activity on skeletal muscle by regulating myoblast activity [49] and miR-150 possesses endogenous haematopoietic activity [50]. The variation is so great that functional miRNAs have been well-established as an evolutionarily advantageous trait [51,52]. The heritability of miRNA and the relationship between chromosomal fragile sites and miRNA density, however, is far more complex than it has been estimated [53]. Kumar *et al.* in 2013 established that miRNA is present in sperm and hence can be inherited by future generations [54]. The role of miRNA in sperm is currently undefined. However, there are many potential areas of reproductive significance that may be influenced by the wide range of miRNA activities. It is worth mentioning that miR-155 has been demonstrated to possess immunosuppressive activity [55] and, therefore, miRNAs may possibly be playing a role in immunosuppression of the maternal immune system, to allow the sperm to reach and fertilize the egg unhampered. 

miRNAs would also be expected to be involved in embryogenesis and differentiation, due to the genetic and epigenetic significance of these events [54]. miR-145 is shown to suppress the pluripotency of human embryonic stem cells [56], which suggests that miRNA expression could prevent the premature occurrence of specific genetic events potentially associated with ectopia and malformations. A potential example of this is the maternal to zygotic transition (MZT) [57], a process that is crucial to mammals. Sperm-derived miRNA, in this context, may be playing a role in the process of regulating maternal mRNA in response to specific co-factors and/or cues, allowing time for synchronous cell division to occur before the midblastula transition (MBT), the interference of which can have devastating consequences.

In zebrafish (*Danio rerio*), Wienholds and co-authors have identified that, instead of tissue fate establishment, miRNAs are more likely to play a role in the maintenance of tissue identity [58]. This suggests an early role of tissue specific miRNA localization and while it does not consider the circulating miR-150 directly, the orthologous expression of miR-150 between humans and zebrafish [59] may make it a good model organism for future studies of intraspecific miR-150 evolution. In light of the evidence mentioned above, it is therefore fair to assume that miRNAs have evolved over time with humans as part of an intraspecific pattern.

## 5. Interspecific Mechanisms of miRNA Evolution and Accumulation as the Species Advances

**Figure 2:**
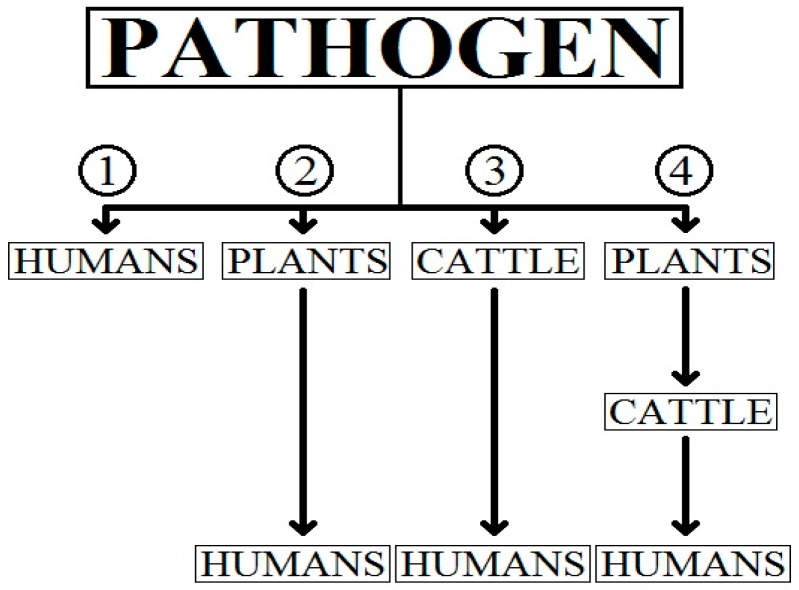
Four possible entry routes of miRNAs from pathogens to humans, labelled 1–4, have been discussed in this review. It is important to identify possible miRNA entry routes to understand the processes of bioaccumulation and overexpression of certain miRNAs such as miR-150.

The discovery of miRNA in sperm effectively overshadowed the previous experimental findings that worms and plants used miRNA in a transgenerational inheritance pattern [60], as it indicated that mammalian miRNAs evolved from other mammals, mimicking their divergent inheritance pattern. There is another seemingly neglected mechanism of miRNA inheritance—which is of paramount importance in the context of miR-150 and oncogenesis—can plant miRNA be recognized and allowed to play epigenetic roles by the mammalian immune system upon oral ingestion? [61]. The discovery that miRNA evolution is linked to complex food web interactions is crucial to our current knowledge, as it involves miRNA in the nutrigenomics of oncogenesis (Figure 2).

The various situations shown in Figure 2 are:

(i) Situation 1 describes the direct uptake of miR-150 from pathogens to humans.
(ii) Situation 2 describes the uptake of miR-150 from pathogens by plants and to humans through oral transfer (eating).
(iii) Situation 3 describes the uptakes of miR-150 by cattle and then humans by consumption of either meat or by the consumption of milk and dairy products [62].
(iv) Situation 4 describes the uptake of miR-150 from pathogens to plants, then by cattle (grazing) and finally humans by considering the food web/trophic levels.

miR-150 is an endogenous miRNA that has physiological roles and functions to provide an adaptive advantage when fully functional and/or regulated [27]. With respect to Figure 2, it is to be hypothesized that dysfunctional miR-150 can be dealt with by coping mechanisms to a certain extent that can be considered as a threshold. When this threshold is exceeded then ontogenetic activity will occur by the saturation of protective factors. The hypothesized evolutionary progression of miR-150, as described in Figure 2, considers four possible scenarios of how humans acquired miR-150 and potentially how humans overexpress miR-150 as a result of bioaccumulation mechanisms. As functional mutations forming in multicellular organisms are unlikely to have created miRNAs, the focus on evolutionary miRNA acquisition will be entirely on that of pathogenic uptake, as unicellular pathogens like viruses have a significantly higher reproductive and evolutionary rate and are therefore significantly more likely to have spontaneously formed miRNA [63]. The possible mechanisms of evolutionary uptake of miRNA-150 from pathogens or vector to humans (categorized as 1, 2, 3 or 4) have been described in Figure 2.

We believe situation 1 will not be possible, as direct pathogen to human interactions are highly unlikely to cause epigenetic changes without chronic exposure, unless in incredibly large concentrations. Even in such a scenario, a person is likely to get a large immune response and potentially die of shock, taking the miRNA out of the gene pool with them, and not transferring the condition through generations.

Situations 2 and 3, are possible routes of excessive miR-150 accumulation as plants and cattle are documented to possess miRNAs, however the quantity of miR-150 from either source alone in causing PCa via bioaccumulation would require vast amounts of the same plant to be consumed which for omnivorous humans, is unlikely to be the case. The requirements for uptake of miR-150 implies that pathogenic effects on the organisms under situation 3 is a possibility, yet highly unlikely, as the immune system of the animal would identify such novel proteins using MHC class 1 molecules and would be quite likely to cause the death of such cells. Furthermore unless gametic cells are affected it is unlikely that such changes would be taken up by future generations of the cattle.

Situation 4 is perhaps one of the most likely pathways for humans to receive excess miR-150, thereby causing health concerns such as PCa. *Agrobacterium tumefaciens* (a potential pathogen), for example, is well established to have oncotic activity in plants, and hence can modify the genome of the plant to integrate miRNA and have downstream trans-generational effects; these plants are, in turn, consumed by cattle, that in essence concentrate the amount of miR-150 by bioaccumulation as was mentioned in situations 2 and 3. A relatively large concentration of miR-150 is therefore taken up after chronic human consumption of animal products such as meats (both, cooked and processed), milk and dairy products such that the endurance threshold is exceeded and miR-150 is deregulated.

While the literature search did not yield specific information on plant species that possess miR-150, Zhang *et al.* in 2011 described the expression of exogenous plant miRNA in the sera and tissues of animals that consumed the plants [64]. Furthermore, rice-derived miR-168 was found to be highly expressed in the sera of Chinese subjects, suggesting that there is a pattern of miRNA acquisition and that the mechanism of uptake and bioacumulation should not be ignored in the context of miR-150 and its potential role as a biomarker for PCa [65]. Sun *et al.* (2015) also identified certain bovine miRNAs while working on miRNA content of bovine milk obtained from lactating Holstein cows challenged with *Staphylococcus aureus* and provided specificity for some of the most commonly identified miRNAs in milk, which may affect downstream gene expression in humans post ingestion [66].

## 6. Pathogens

The role of various possible pathogens is important to know. This is especially important because in order to identify a possible source and role of miR-150 in causing diseases in human it is vital to understand if host immune response due to infection(s) changes the overall contents and expression of certain miRNAs in exosomes.

Parasites and fungi are an unlikely source of genetic modifications, as the immune system is primed to destroy these pathogens and the infected cells. These organisms have moderate evolutionary rates and, like bacteria, have many mechanisms for preventing mutation but can cause epigenetic changes to hosts [67]. 

Bacteria are a possible source of genetic modifications. For example, *Agrobacterium tumefaciens* and *Helicobacter pylori* which are known to have the ability to infect and cause crown gall in plant cells [68] and gastric cancer in humans and other animals [69], respectively, have both relatively high evolutionary rates, but have many mechanisms for preventing mutations. 

Viruses, however, are perhaps the most likely potential source of miRNAs, especially retroviruses which make up 8% of the human genome predominantly by transposons [70], which intriguingly also contain hairpin structures. Retroviruses integrate their base sequences into their host genome and have short reproductive phases resulting in large amounts of mutation and variation making them ideal candidates. Furthermore, retroviruses like the human immunodeficiency virus (HIV) require a high viral load to cause advanced infection and acquired immune deficiency syndrome (AIDS).

Thus, it is definitely possible that miRNAs from pathogen sources, bacterial and viral in particular, have an important role to play in the expression of certain diseases due to their capability to undergo interspecific transfer. Two studies have recently been carried out by Lawless *et al.* (2013) to analyze the expression level changes of miRNAs in response to Gram-positive bacterial infection of a bovine cell line [71] and on bovine monocytes from the milk and blood of cows infected with *Staphylococcus uberi* [72]. These results suggest that our hypothesis regarding the change in miRNA expression due to pathogenic infection of food sources having an impact on gene expression in the humans after ingestion has already been looked into and holds true in a mammalian system.

## 7. Nutrition

With the shift in focus of therapy from treatment to prevention, diet has come under increasing scrutiny, especially with the findings of Spisak *et al.* (2013) that identified the presence of foreign nucleic acid in human plasma [73]. If plants have minute quantities of potentially carcinogenic miRNAs and cattle are known to take up ingested plant miRNAs [74], then meat and dairy products in particular are of dietary importance due to the plausible bioaccumulation of the potentially oncogenic miRNAs through human dietary consumption. 

Furthermore, cooking methods may also have a role in the amount of miRNA remaining in the animal tissue consumed. In managing pernicious anaemia, raw liver was found to contain vitamin B12 (intrinsic factor), which decreased (but was not entirely eliminated) when cooked [75]. This finding encourages us to suggest that undercooked or raw red meats are therefore likely to have more miRNAs while more cooked samples could have decreased amounts as the miRNAs are degraded.

In the literature surrounding nutrition, there has been a recent surge in evidence for the carcinogenicity of red meats [76,77]. The reason behind this conclusion was that the process of cooking the meat released volatile compounds (*i.e.*, N-nitroso compounds) that play important roles in mutagenesis and carcinogenicity [76,77]. With the previous considerations however, miRNA provides an alternate potential mechanism of oncogenesis by suggesting that the lack of miRNA clearance could also contribute to oncogenesis.

## 8. Mechanism of Action

As proposed earlier, miRNA is dysregulated in oncogenesis potentially as a result of dietary alterations to concentration levels. This mechanism requires an understanding of the pathological process of miR-150 in the context of cancer in order to be applicable to future treatments, for which, we forsee there is a lot of potential. miR-150 is a miRNA implicated in a wide variety of cancers including breast cancer [78], colorectal cancer, osteosarcoma and most notably PCa. 

Interestingly, the role of miR-150, however, varies in its contribution to each cancer. In breast cancer its overexpression decreases apoptosis and enhances growth [22]; in contrast, in hepatocellular carcinoma, the overexpression of the *ZFAS1* gene and consequent binding of ZFAS1 protein to miR-150, prevents it from exerting its contrasting tumor suppressive effects [65]. 

miR-150 should therefore be examined in a cancer-specific manner and, thus, based on the various arguments outlined above, the focus should consider the effect of miR-150 on PCa. PCa is a very common cancer, particularly in ageing western populations of developed nations [79], although there are no conclusive experiments that indicate a direct mechanism of initiation and progression of the disease. miR-150, however, can be used as a potential biomarker both at the diagnostic level and at therapeutic level, because it can be readily accumulated via consumption and is associated with malignancy. Therefore, it would be interesting to knock miR-150 down by using molecular tools and assess its effects on PCa cells.

## 9. Conclusions

This is a hypothesis-driven manuscript, so we have had our limitations in terms of finding desired published literature. We, however, think that there is a possibility of moving towards the identification of a non-invasive diagnostic biomarker for PCa and/or aggressive PCa by quantifying circulating miR-150. The core idea of miR-150 being bioaccumulated and interspecifically transferred is also vital for the identification of a possible therapeutic biomarker for PCa, as we believe that we are what we eat. 

However, one of the major limitations of this hypothesis when doing experimental research will be to define the specific required quantity of miR-150 in a healthy human being. Nevertheless, we believe that miR-150 has undoubtedly a lot of potential to be developed as a specific cancer biomarker in the near future.
