# Correction: Vaidyanathan *et al*. Are We Eating Our Way to Prostate Cancer—A Hypothesis Based on the Evolution, Bioaccumulation, and Interspecific Transfer of miR-150. *Non-Coding RNA* 2016, *2*, 2.

**DOI:** 10.3390/ncrna2020006

**Published:** 2016-06-20

**Authors:** Venkatesh Vaidyanathan, Vetrivhel Krishnamoorthy, Nishi Karunasinghe, Anower Jabed, Radha Pallati, Chi Hsiu-Juei Kao, Alice Wang, Gareth Marlow, Lynnette R. Ferguson

**Affiliations:** 1 Discipline of Nutrition and Dietetics, FM & HS, University of Auckland, Auckland 1023, New Zealand; vkri468@aucklanduni.ac.nz (V.K.); rpal628@aucklanduni.ac.nz (R.P.); b.kao@auckland.ac.nz (C.H.-J.K.); alice.wang@auckland.ac.nz (A.W.); l.ferguson@auckland.ac.nz (L.R.F.); 2 Auckland Cancer Society Research Centre, Auckland 1023, New Zealand; n.karunasinghe@auckland.ac.nz; 3 Department of Molecular Medicine and Pathology, FM & HS, University of Auckland, Auckland 1023, New Zealand; a.jabed@auckland.ac.nz; 4 Experimental Cancer Medicine Centre, Cardiff University, Cardiff, CF14 4XN, UK; MarlowG@cardiff.ac.uk

The authors wish to make the following correction to this paper [1]. Reference 28 has been renumbered to 29, and the following affirmation in the original text: “Dezhong *et al.* have demonstrated that miR-150 was overexpressed in PCa cells at both the mRNA and protein levels” has been substituted by: “Waltering *et al.,* 2011, reported that miR-150* along with three other miRNAs, namely, miR-10a, miR-141, and miR-1225-5p, among 17 miRNAs, expressed to have >1.5- fold changes at the expression levels in PCa cells and in a minimum of two intact-castration xenograft pair. It has been shown that miR-150 was overexpressed in PCa cells.”

Thereby, we include the research done by another group—Waltering *et al*., 2011—who also found interesting results working on PCa cell-lines and xenografts and the effect of miRNAs (reference no. 31 in the edited version).

References 29 and 30 were initially cited unintentionally on page 2 of the article. These references have been deleted from page number 2 and renumbered to 38 and 39 on page 3.
